# Microfabricated, amperometric, enzyme-based biosensors for in vivo applications

**DOI:** 10.1007/s00216-016-9420-4

**Published:** 2016-03-02

**Authors:** Andreas Weltin, Jochen Kieninger, Gerald A. Urban

**Affiliations:** Laboratory for Sensors, Department of Microsystems Engineering – IMTEK, University of Freiburg, Georges-Köhler-Allee 103, 79110 Freiburg, Germany

**Keywords:** In vivo, Biosensor, Chemical sensor, Microfabrication, Glutamate, Lactate

## Abstract

Miniaturized electrochemical in vivo biosensors allow the measurement of fast extracellular dynamics of neurotransmitter and energy metabolism directly in the tissue. Enzyme-based amperometric biosensing is characterized by high specificity and precision as well as high spatial and temporal resolution. Aside from glucose monitoring, many systems have been introduced mainly for application in the central nervous system in animal models. We compare the microsensor principle with other methods applied in biomedical research to show advantages and drawbacks. Electrochemical sensor systems are easily miniaturized and fabricated by microtechnology processes. We review different microfabrication approaches for in vivo sensor platforms, ranging from simple modified wires and fibres to fully microfabricated systems on silicon, ceramic or polymer substrates. The various immobilization methods for the enzyme such as chemical cross-linking and entrapment in polymer membranes are discussed. The resulting sensor performance is compared in detail. We also examine different concepts to reject interfering substances by additional membranes, aspects of instrumentation and biocompatibility. Practical considerations are elaborated, and conclusions for future developments are presented.

**Graphical Abstract:**
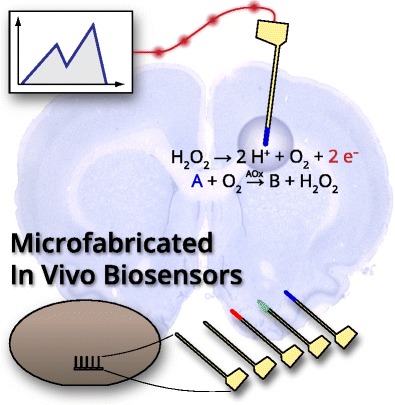
ᅟ

ᅟ

## Introduction

The measurement of metabolic parameters by microsensors directly within the tissue can be a valuable tool in biomedical research and holds much promise for clinical practice. Unlike the measurement of electrical signals in neural recording, modified microelectrodes can be used as chemical sensors to detect concentrations of chemicals such as energy metabolites or neurotransmitters. These chemical sensors are biosensors if enzymes with their very specific interaction with a substrate are immobilized as biological recognition elements onto such electrodes. This method allows the fabrication of selective, amperometric electrochemical sensors, where a potential is applied and the measured current is proportional to the analyte concentration. Microsensors are self-contained, chemical and biosensor devices miniaturized to functional dimensions of typically tens to hundreds of micrometres, and have an electrical output signal in our case.

The most widely studied in vivo sensors are continuous glucose monitoring systems aimed at patients with metabolic disorders (e.g. diabetes). However, they have been covered extensively in the literature, in terms of both theory and practical applications [1–3] and will therefore not be the focus of this article. The other main driving force behind in vivo sensors is the neurosciences. Many microsensors have been developed for short-term brain application in animal models, mostly for neurotransmitters (e.g. glutamate or choline) and energy metabolites (e.g. lactate). Here, the focus is shifted more towards basic biomedical research. Application has been limited to animals so far, in contrast to the use of continuous glucose monitoring systems in clinical or personal medicine.

Electrochemical sensor systems are easily miniaturized and fabricated with microfabrication technologies. Small, reproducible electrode geometries on thin, needle-type probes can be achieved, resulting in a high spatial resolution and minimal sensor footprint. A number of powerful fabrication tools used in microsystems engineering are available. Enzyme-based, amperometric, electrochemical biosensing allows a highly selective and sensitive response in a complex environment. In contrast to other techniques such as microdialysis and nuclear magnetic resonance (NMR) spectroscopy, microsensors can rapidly and precisely measure extracellular low analyte concentrations within the tissue in near real time. From both an engineering point of view and a sensor development point of view, we discuss sensor principles and microsensor platforms. Because of the countless different biosensor principles, ever-evolving electrode materials and electrochemical methods, we focus on those concepts that have been successfully transferred to self-contained microsensor platforms for actual in vivo use. Non-enzymatic in vivo sensors (e.g. for the detection of the monoamine neurotransmitters dopamine and serotonin, where the analyte is electroactive and can be converted directly at the electrode) are not discussed either [4–7].

In this article, we describe and compare different platform and sensor integration technologies for enzyme-based amperometric in vivo microsensors, discussing work relevant to the current state of the art regarding fabrication and performance. We compare the microsensor approach with other techniques to illustrate where such sensors can be applied, what advantages can be expected and how they complement other methods. We describe the different microfabrication techniques for amperometric in vivo sensors, ranging from simple modified wires and fibres to fully microfabricated systems on silicon, ceramic or polymer substrates. We discuss recent developments (e.g. in silicon and flexible microtechnology). The various immobilization methods for the enzyme are compared, as are their implications for sensor performance, where a detailed literature review is included. We also describe different concepts to reject interfering substances, to provide selectivity and to provide biocompatibility. Practical considerations are elaborated and conclusions for future developments are presented.

## Background

### Metabolism and relevance of measured parameters

#### Glutamate and neurotransmitters

Glutamate is well known as the main excitatory neurotransmitter in the central nervous system (CNS). It is involved in many aspects of neuronal functions and diseases thereof. In a simple description, glutamate and other neurotransmitters are stored inside neurons in relatively high concentrations, around 10 mM in the case of glutamate [8, 9]. On activation of neurotransmission, they are quickly released into the synaptic cleft, where they propagate the signal across the synapse. To maintain a high signal-to-noise ratio of the synaptic transmission, neurotransmitters are cleared from the extracellular space rapidly by efficient uptake mechanisms. Glutamate is taken up mainly by astrocytes, converted to glutamine and shuttled back to the neurons. The physiological extracellular background concentration for neurotransmitters in the CNS is only in the low micromolar range. This difference from the intracellular value serves as a good example why volume methods such as NMR spectroscopy measure entirely different concentrations compared with measurements in the extracellular space. Elevated extracellular glutamate concentrations also lead to neuronal death. From the dimensions of the synaptic cleft (300 μm width, 20 nm gap [10]) and the timeframe (milliseconds [11]) in which high concentrations (1 mM [10, 11]) are present there, it is clear that current amperometric microsensors cannot aim at measuring “synaptic” neurotransmitters, but rather can aim at changes in the extrasynaptic background. Neurotransmitters also have a multitude of functions beyond synaptic neurotransmission [9]. Furthermore, the glutamate–glutamine cycle is the main energy consumer in the CNS, underlining the relationship between energy and neurotransmitter metabolism [12, 13].

#### Energy metabolites

Glucose as the primary energy source for cells is normally supplied to most tissues through the bloodstream in relatively high and stable concentrations (4–6 mM), especially the brain (1–3 mM) [14]. Lactate is the main product of anaerobic metabolism. Besides being a direct indicator for the amount of anaerobic metabolism, it is more than a metabolic waste product. It can act as a neuroprotector and under certain conditions as an energy substrate [15]. Especially the lack of oxygen (e.g. because of stroke or trauma) can lead to the breakdown of neurotransmitter metabolism, with understandably severe consequences. In an ageing society, ischaemic stroke has an increasingly larger share among the causes of death and acquired disabilities with a high socioeconomic relevance [16, 17]. A focus of research is also the battle against the numerous neurodegenerative diseases, where alterations in neurotransmitter metabolism are often observed [18]. It is therefore quite obvious that the fast, precise and extracellular measurement of metabolic parameters is of interest in fields ranging from fundamental biomedical research to clinical intensive care. Although the measurement of neurotransmitters outside the CNS does not play a large role, that of energy metabolism does. Changes in cellular metabolism are closely linked to cancer. Inside tumours a hypoxic environment with a highly anaerobic metabolism is often found [19, 20]. Any disruption in blood supply and thus oxygen and energy supply (e.g. during surgery, transplantations, sepsis or trauma) will lead to alterations in metabolism. Here another field of activity for microsensors may evolve in the future.

#### Enzyme-based amperometric microbiosensors

**Fig. 1:**
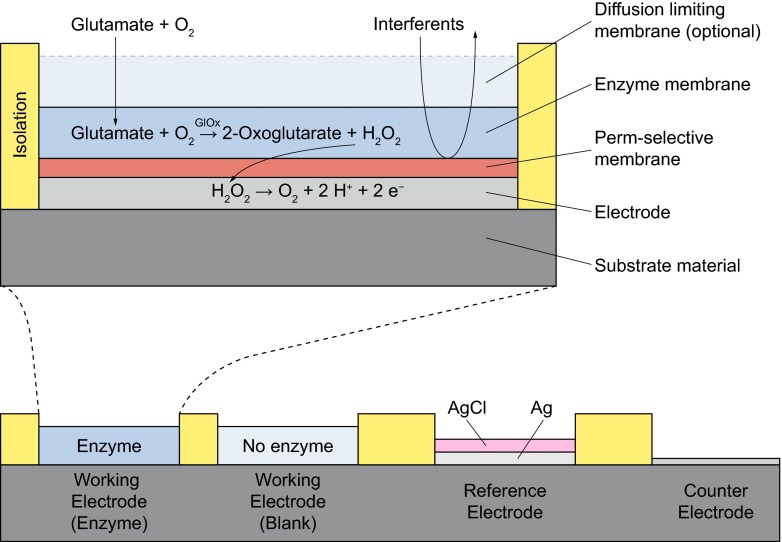
Enzyme-based, first-generation biosensor principle on microelectrodes with glutamate as an example and a typical planar electrode arrangement as found in microsensors. The analyte diffuses into the membranes together with oxygen. The enzyme is immobilized in the enzyme membrane and converts the analyte to hydrogen peroxide. The hydrogen peroxide diffuses to the electrode, where it is oxidized at the appropriately polarized electrode, generating the measured current. Interferents are held back by the permselective membrane and cannot be oxidized at the electrode, generating no additional signal. The diffusion-limiting membrane can limit the transport of the analyte in relation to the oxygen transport if the linear range has to be extended. The working electrode contains the enzyme and measures the selective signal. The blank electrode is similar only without immobilized enzymes. It measures only the unspecific background, and its signal is subtracted from that of the working electrode. Electrodeposited Ag/AgCl can serve as a pseudo-reference electrode for the polarization of the working electrode. *GlOx* glutamate oxidase

Enzyme-based amperometric microbiosensors are typically realized by a set of immobilized membranes on noble metal or carbon-based electrodes (Fig. 1). In a first-generation biosensor, an enzyme converts the analyte to an intermediary product, which is then oxidized at the electrode [1]. The resulting current flow is proportional to the analyte concentration. The enzyme proteins have to be immobilized in a matrix or membrane without their functionality being lost. Most commonly, these are oxidase enzymes (e.g. glucose oxidase [21–23], lactate oxidase [21, 24–26], glutamate oxidase [26–29] or choline oxidase [29, 30]), which convert their respective analyte to hydrogen peroxide under the consumption of oxygen as a natural co-substrate (Fig. 1). Hydrogen peroxide can be efficiently oxidized at polarized noble metal electrodes (e.g. platinum) in a two-electron process [31]. Since other biogenic substances can also be oxidized at the electrode, it is often covered with a permselective membrane, which prevents other substances from reaching the electrode [32].

One disadvantage of this sensor principle is the dependency on oxygen, which has limited solubility in aqueous fluids, only approximately 200 μM under physiological conditions. It is always available only in lower concentrations within tissues because of restricted supply by blood vessels and permanent cellular consumption. Therefore when one is measuring higher analyte concentrations, the aim is to achieve a diffusion-limited regime by limiting analyte diffusion with another membrane. Ideally, this membrane limits the transport of oxygen to a much lesser degree than analyte diffusion, and thus allows an unaltered enzymatic reaction even at high analyte concentrations. For example, to fully convert 20 mM glucose, its diffusion must be limited by a factor of 100 with the mentioned oxygen concentration because of the 1:1 stoichiometry of the enzymatic reaction. The enzyme is typically loaded into the enzyme membrane in high concentrations so that the sensor performance is not kinetically limited by the enzymatic reaction but by diffusion of the analyte only. Oxygen dependency may not affect the measurement of low-concentration neurotransmitters, but it is crucial for glucose or lactate monitoring.

With the goal to eliminate oxygen dependency, second-generation, reagentless biosensors use an additional electron acceptor (mediator) as a substitute for oxygen [1]. In this principle, the enzyme is reduced and the substrate is converted to the product. Then, the reduced form of the enzyme reduces the mediator and is in turn oxidized by an intermolecular electron transfer. In the final transduction step, the mediator is oxidized at the electrode, resulting in electron transfer to the electrode. Redox polymers can contain both an enzyme and a mediator [33, 34]. In third-generation biosensors, the electron transfer from the enzyme to the electrode occurs directly, without mediators or a co-substrate but with often limited efficiency of the electron transfer [1].

Microfabrication technology allows the efficient, parallel fabrication of small sensor devices [35]. Metal microelectrodes can be precisely structured on silicon [29], ceramic [28] or polymeric [21] substrates by lithography. Large numbers of electrodes can be modified in parallel by electrodeposition processes. These may include the deposition of silver and its conversion to silver chloride (AgCl) for the integration of a pseudo-reference electrode. The deposition of membranes, with or without enzymes, as liquids with subsequent cross-linking can be performed precisely and in low volumes (microlitre to nanolitre range) by dispensing or coating processes. Finally, individual devices can be shaped by wafer-level etching [29] or automated cutting [26]. An overview is given in Table 1, and the different sensor systems are discussed in “Amperometric microsensor platforms”.

**Table 1:** Overview of selected electrochemical amperometric microsensor platforms for in vivo measurement, including substrate type/material, electrode material, realized sensor parameters and applied sensor immobilization/interference rejection methods

Substrate	Electrode	Parameter	Sensor immobilization	Interference rejection	References
Wire/disc	Pt/Ir	Glu, L	BSA + GA	Nafion, AAOx	[24, 27]
	Pt/Ir	Glu, G, L, Ch	BSA + GA	Nafion, AAOx	[36, 37]
Ceramic	Pt	Glu, L, Ch, ACh, Ad	BSA + GA	PPD, Nafion	[25, 28, 38, 39]
Carbon fibre	C	Glu	Redox hydrogel	Nafion, AAOx	[33]
	C	Glu	Redox hydrogel	Nafion, AAOx	[34, 40]
	C/Ru	G, L, Glu	BSA + GA	PPD, Nafion	[22]
	C	L	BSA + GA	CA	[41]
Silicon	Pt	Glu, Ch	BSA + GA	mPD	[29]
	Pt	Glu	BSA + GA	PPy, Nafion	[42, 43]
	Pt	G, L	BSA + GA, PEGDE	PPD	[23]
Polyimide	Pt	G, L, Glu, O_2_, pH	PHEMA, BSA + GA	PPD	[21, 26, 44]

*AAOx* ascorbate oxidase, *ACh* acetylcholine, *Ad* adenosine, *BSA* bovine serum albumin, *CA* cellulose acetate*, Ch* choline, *G* glucose, *GA* glutaraldehyde, *Glu* glutamate, *L* lactate, *mPD m*-phenylenediamine, *PEGDE* poly(ethylene glycol) diglycidyl ether, *PHEMA* poly(hydroxyethyl methacrylate), *PPD* polyphenylenediamine, *PPy* polypyrrole

#### Instrumentation

The output value of an amperometric sensor is the current for an applied constant potential. Thus amperometric sensors are operated in electrochemical non-equilibrium. The simplest case is the polarization of two electrodes, a working electrode and a combined reference/counter electrode. In many cases, electrochemical in vivo sensors are operated this way [23, 24, 42, 45]. However, a combined reference/counter electrode should be substantially larger than the working electrode to show a potential independent of the current. On one hand, such an electrode should be involved in a redox reaction with high exchange current density to ensure a stable potential, whereas on the other hand, only inert electrodes prevent change or even consumption of the electrode material due to the sensor current. Therefore microsensors should preferably use a three-electrode setup with a separated reference electrode and counter electrode [22, 26, 29]. The reference electrode (e.g. Ag/AgCl) is kept currentless, whereas the counter electrode can be inert (e.g. Pt).

In such three-electrode setups, the voltage between the working electrode and the reference electrode is regulated to a set value by a current flowing through the working electrode and the counter electrode. The current is the sensor output and represents in the ideal case the conversion of the analyte only. This type of circuit (and often also the corresponding device including a data acquisition unit) is called a “potentiostat”. To measure low current levels, two possible influences of electrical noise have to be considered: one influence is on the stability of the feedback loop in the regulation circuitry and the other one is a noisy output signal. The first influence is more crucial and has to be dampened appropriately in the analogue circuit. As a worst case, the working electrode is polarized with an inappropriate potential. From the perspective of measurement technology, it would be favourable if the potentiostat circuitry (or at least the analogue part) were in the direct vicinity of the sensor electrodes. For in vivo experiments, sometimes headstages are used, preamplifiers mounted directly on the animal [45–47], mostly to reduce noise in the connection lines in freely moving animals. If the device is remote, all the wiring is within the feedback loop of the potentiostat circuitry and is therefore sensitive to noise, which may cause instability. In amperometry with constant potential or chronoamperometric protocols using stepwise constant potentials to precondition electrodes, the circuits do not have to be faster than the acquisition rate. That is especially helpful for low signals in a noisy environment because the bandwidth of the feedback loop can be limited to low frequencies.

Several approaches to fabricate integrated potentiostats have been described in the literature [48–51]. There are some integrated circuits providing potentiostat functions available on the market (LMP 91000, Texas Instruments). However, to our knowledge, there are no reports of the application of such devices in an in vivo experiment. The integration of circuits into the sensor device is, however, in contradiction to the concept of disposablity behind microsensors.

### Alternative methods

In the discussion of the use of microsensors in vivo, the question arises if the use of such an invasive method can be justified, and what particular advantages are to be expected. Certainly, a number of alternative techniques to measure metabolites in vivo are available, with many of them being used widely in clinical practice (e.g. microdialysis or NMR methods). However, they differ, often substantially, regarding what in the tissue is actually measured and where, and what analytical performance can be expected. Thus comparison with other techniques (summarized in Table 2) not only helps to select the right approach for the application but also helps to point out and discuss the advantages and limitations of microsensor measurement.

**Table 2:** Comparison of different methods for in vivo measurement, comparing the invasive microsensor and microdialysis methods with the non-invasive nuclear magnetic resonance, positron emission tomography and fluorescence imaging techniques

Method	Advantages	Disadvantages
Microsensors	High temporal resolution (<1 s) Low detection limit (<1 μM) High spatial resolution (<100 μm) High precision Extracellular measurement	Invasiveness Limited lifetime Limited number of analytes
Microdialysis	Low detection limit (<1 nM) Large number of analytes Powerful detection method High precision Extracellular measurement	Invasiveness Low temporal resolution (delay of seconds to minutes) Fluidic setup necessary
Nuclear magnetic resonance	Non-invasiveness Direct detection of chemical structure Large number of analytes	Low precision (mM) High detection limit (mM) Low temporal resolution (more than minutes) Low spatial resolution (cubic millimetres to cubic centimetres) Integral extracellular and intracellular values Costly, large equipment
Positron emission tomography	Non-invasiveness (except tracer) Low detection limit (<1 μM) Large number of analytes	Radioactive exposure Tracer necessary Low temporal resolution (seconds to minutes) Low spatial resolution (mm^3^) Costly, large equipment
Fluorescence imaging	Low detection limit (<1 nM) High temporal resolution (ms) High spatial resolution (<1 μm)	Indirect via markers Complex engineering of markers Optical access needed

#### Microdialysis

One of the most widely used in vivo techniques is microdialysis [52, 53]. This fluidic method is used to sample and extract fluids from tissues for external analysis. It has been used frequently in animal models and humans, and also in clinical applications—for example, traumatic brain injuries [54, 55]. In this invasive method, a probe is inserted into the tissue, through which a fluid, the perfusate, is pumped. An exposed semipermeable membrane allows the exchange of analytes between the tissue and the fluid. The perfusate with the analytes, the dialysate, is guided outside the tissue and analysed externally, with a number of both online and offline methods. In practice, these minimally invasive probes are, for example, double hollow fibres of around 250–500-μm diameter and several millimetres long, where the perfusate enters in the inner fibre and the dialysate flows back in the outer lumen. The semipermeable membrane, with a cut-off of 5–50 kDa, is exposed at the tip of the probe.

The efficiency of the extraction of the analyte from the tissue depends on a number of factors, such as the probe and membrane geometry, the flow rate of the perfusion and the total sample volume. Because of the diffusion through the membrane, pumping and external analysis, this method always has a time delay. One of the drawbacks of microdialysis is that the higher flow rate and the lower the membrane area are, the higher the temporal resolution and the spatial resolution become; however, the concentration of the analyte in the dialysate becomes lower as it is entirely dependent on its diffusion, and at zero flow, the analyte concentration in the dialysate can become equal to the tissue concentration at best. Novel fluidic methods such as use of discrete droplets and segmented flow improve sample collection [56, 57]. Separator fluids or gas bubbles segment the perfusate into nanolitre-range droplets to prevent diffusion out of the small confined volume. Zero-flow protocols can be applied in this way without analyte dispersion.

In comparison with the in situ measurement with sensors, a large number of powerful detection techniques are available for external analysis of microdialysates. Offline methods such as high-performance liquid chromatography or mass spectrometry offer superior analytical performance. That allows the identification of a large number of different analytes with high precision and low detection limits. However, offline analysis results in additional time delays and does not allow continuous monitoring. Online methods include use of electrochemical sensors and capillary electrophoresis. Yet, the time delay even for rapid systems is still non-negligible. For electrochemical sensors it ranges from 30 s to a few minutes [58, 59]. For online capillary electrophoresis with laser fluorescence detection, a temporal resolution of 12 s was reported for glutamate measurement in animals [60]. A rise time of 60 s was achieved by on-chip electrophoresis with amperometric detection [61]. Still, the fast dynamics of some neurotransmitters in combination with low concentrations are impossible to measure by microdialysis. Additionally, the measurement of dissolved gases with high diffusivities such as oxygen is very cumbersome with external microdialysis methods because of high out-diffusion during transport.

#### Nuclear magnetic resonance

Especially in the neurosciences, NMR techniques are used not only for imaging but also to determine tissue concentrations of metabolites. The advantage is the non-invasiveness. On the basis of the alignment of nuclear spins in a strong magnetic field and the absorption and reemission of non-ionizing electromagnetic radiation, NMR spectroscopy uses frequency spectra to identify chemical compounds by their specific chemical structure. Besides measuring protons for imaging, this method also allows the direct detection of chemical compounds. One difficulty here is the separation of structurally related substances such as glutamate and glutamine, which becomes harder at a lower magnetic field strength [62]. The method can be enhanced by the labelling of molecules with the isotope ^13^C, which often has to be injected because of its low natural abundance and metabolized into the compounds first [63, 64].

With regard to measurements in tissue, the principal difference between NMR spectroscopy and electrochemical sensing/microdialysis is that NMR spectroscopy is a volume-based method, which cannot discriminate between extracellular and intracellular concentrations. For neurotransmitters, where the difference between the two concentrations is often particularly high, this limitation is consequently severe. The regions of interest (voxels) that can be resolved are rather large, usually in the range of 1–100 ml, compared with the size of microelectrodes, [65–67]. The time to acquire precise spectra is at least in the range of a few minutes, limiting the temporal resolution of the method, with there being a trade-off between sensitivity and temporal resolution. Exact analytical performance is rarely ever discussed and calibrations are often not shown. Compared with electrochemical sensors, data suggest high detection limits, poor selectivity and low precision, with high deviations of 10 % reported [68]. This drawback is supported by the lack of studies of the less-abundant substances present in concentrations lower than the millimolar range. Thus, NMR methods are more suitable to study long-term distribution, metabolic cycling and storage of metabolites rather than fast transient changes at low concentrations, which is usually the case for at least neurotransmitters.

#### Positron emission tomography

Another clinically relevant method is positron emission tomography (PET). Radioactive tracer substances are introduced into the tissue, from which the emitted gamma radiation is then detected. Species such as ^15^O or fluorodexoyglucose (FDG), where ^18^F is bound to a glucose molecule, are used as tracers. Apart from the tracer itself, the method is non-invasive. The disadvantages of the method are that tracers with limited availability and lifetime have to be administered to the organism first and, in the case of FDG, metabolized, which takes at least several minutes. FDG is taken up into cells but unfortunately not metabolized further. The reported spatial resolution is in the range of 2.5–3 mm [69, 70], limited by the physical principle, and the acquisition rates are approximately one measurement per minute [71]. For mapping regional cerebral oxygen distribution (e.g. after stroke) ^15^O PET is considered a standard. Dopamine can be measured by PET because its precursor dihydroxyphenylalanine can be labelled with ^18^F. Furthermore, performance is dependent on the local concentration and the binding of the tracer to the target molecule. New tracers are under development for mapping in the context of neurodegenerative diseases [72].

#### Optical methods

Optical and fibre optical (bio)sensors, enzymatic and non-enzymatic, based on fluorescence or spectroscopy, including numerous different transduction principles, have been reviewed in detail elsewhere [73–75]. Few miniaturized systems for in vivo application outside the field of glucose monitoring have been realized, possibly because of the lack of appropriate fluorescent dyes for the desired analytes. Still, it is very likely that optical sensors will play a larger role in the future. Particularly interesting are approaches measuring non-invasively through the skin (e.g. direct near-infrared sensors), although they are not applicable everywhere, especially not in the brain, and are impaired by changing optical path length and interferences.

Especially for glutamate in the neuroscience context, so-called sensors based on fluorescence markers in the tissue have emerged [76–79]. These markers are introduced into the cells and feature a glutamate-binding protein along with a fluorescent protein. Evaluation by multiphoton fluorescence microscopy yields astonishing analytical performances with millisecond temporal resolution, areas of interest in the submicrometre range and submicromolar detection limits [80]. The drawbacks lie in the complex engineering of the marker into the organism by genetically encoding protein alterations, the limited dynamic range due to the non-linear fluorescence signal and expression of the marker, and the need for optical access.

## Amperometric microsensor platforms

### Wires and fibres

A simple way to shape microelectrodes is to use modified wires and fibres, which provided many opportunities when other microfabrication processes were not available. Wires are often insulated by melting glass, capped off and then polished to be used as microelectrodes. Other insulation materials such as polymers can simply be removed for a defined length to expose the entire wire cylinder. Carbon fibres are fixed inside an insulator (e.g. a glass capillary) and then capped off at the tip.

**Fig. 2:**
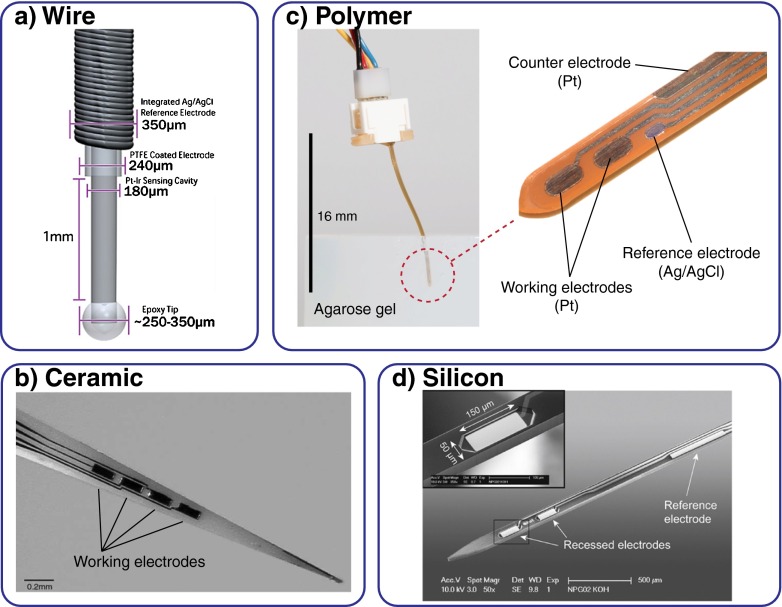
In vivo microsensor platforms. **a** Modified wires or fibres can be exposed and enzymes immobilized on them [36]. Microfabrication technologies allow a more efficient, advanced production and sensor integration. Multiple, small, recessed electrodes with any geometry and layout can be realized. Parallel electrodeposition of membranes and reference electrodes is possible. Fabrication on **b** ceramic [45], **c** polymer [26] or **d** silicon substrates [29] for various sensor shapes has been demonstrated. (All images reprinted with permission from Elsevier)

Hu et al. [24, 27] introduced a platinum wire for lactate and glutamate measurement in the rat brain. The principle and technology have since been commercialized by Pinnacle Technology. The Teflon insulation around a platinum iridium wire of 170 μm diameter was removed for 1 mm length. On protection of the tip by epoxy, a 700-μm-long sensor cavity around the exposed wire was formed. Stability was sufficient for stereotaxic placement in the brain, but the sensor was strengthened by its being glued into a glass capillary after 8–12 mm. Later variants by Pinnacle Technology are almost identical and include a chloridated silver wire wound around the sensing wire as a Ag/AgCl pseudo-reference electrode [36, 37] (Fig. 2a). The group of O’Neill used Teflon-insulated 60–250 μm platinum wires for their glucose [81] and glutamate [82, 83] sensors based on immobilization of the enzyme in an electropolymerized layer. They also compared platinum with other electrode materials, such as gold, palladium and glassy carbon [84].

In a similar fashion, carbon fibres were used not only for direct measurement of, for example, dopamine, but also for enzyme-based electrochemical detection in vivo. A 30 μm carbon fibre exposed for 500 μm and fixed with epoxy into an insulating pulled glass capillary was used by Shram et al. [41] as a lactate sensor. Detection was by hydrogen peroxide oxidation. The enzyme lactate oxidase was deposited by dip-coating. Schuvailo et al. [22] coated a fibre with ruthenium to fabricate oxidase-based glutamate, lactate and glucose sensors. In vivo demonstration was limited however. The bienzyme approach for 10-μm-diameter carbon fibres was used by Kulagina et al. [33] and Oldenziel et al. [34, 85] for measurement of glutamate and ascorbate in the rat brain. For reference, a comparable setup of 5-μm-diameter carbon fibres, exposed for 50–100 μm, fixed in glass capillaries was used to non-enzymatically measure dopamine inside the brain of rats [6].

The simplicity of fabrication has led to the wide use of fibres and wires as in vivo sensors. Multielectrode arrays with defined geometries are however difficult to realize (e.g. by bundling of fibres) in comparison with sensors fabricated with planar microtechnology. Manufacturing of sensors from fibres and wires can be achieved with low-cost equipment, but efficient, parallel mass fabrication is difficult or labour intensive. Enzyme immobilization has been restricted mainly to dip-coating, which allows only limited spatial control and impairs the realization of multianalyte platforms or blank electrodes. Sensor geometry is determined by the fibre/insulation dimensions, and therefore restricts the overall length and the implementation of more complex geometric shapes.

### Silicon-based platforms

The advancement of microfabrication technologies has translated increasingly into the development of silicon-based electrochemical microsensors. Precise structuring of a large number of small electrode geometries on thin, needle-shaped devices is possible, promising high spatial resolution and minimal tissue damage. Besides the development of biosensors, it has led even more to substantial developments of devices for electrical recording from the CNS [86, 87]. Thin noble metal layers can be deposited onto silicon wafers by physical vapour deposition or sputtering and then structured by lithography and a lift-off process. As insulation layers, different combinations of silicon oxide or nitride are available, usually deposited by chemical vapour deposition processes, with possible plasma enhancement. Etching processes, such as reactive ion etching, allow not only the creation of an electrode and contact pad opening through the insulating layer but also the shaping, thinning and release of the entire needle-type structures in the wafer plane (e.g. by deep reactive ion etching). Inconveniences are the need for costly equipment and clean-room facilities for fabrication as well as the brittleness of silicon, which limits the overall device length and robustness.

A silicon process was used by Frey et al. [29] for the fabrication of glutamate and choline microsensors for the brain of rats (Fig. 2d). The silicon needles were 30–100 μm thick, 100 μm wide and 6.5 mm long, with the two working electrodes located at the tip, and a Ag/AgCl reference electrode was integrated further behind. The wafer-level processing included the physical vapour deposition of platinum as the electrode material. Insulation was by provided by nitride deposited by chemical vapour deposition. Thinning from the back side was by deep reactive ion etching, and shaping was done from the front side. The electrodes recessed into the nitride were of size 50 μm × 150 μm. A single shaft or combs of four shafts were connected by wire bonding. An almost identical approach was pursued by Wassum et al. [43], also for glutamate sensors. The probes were 150 μm thick and 120 μm wide and four 40 μm × 100 μm working electrodes were integrated. A reference electrode made from electrodeposited iridium oxide was later integrated [42]. Another silicon platform for the simultaneous recording of glucose and lactate in the brain was introduced recently [23]. Platinum was deposited by physical vapour deposition and 6-mm-long microneedles with a 3 mm implantable part of 100 μm width and 50 μm thickness were shaped by deep reactive ion etching from both sides. Insulation was achieved by the photopatternable epoxy resist SU-8, omitting the need for additional oxide/nitride deposition. This layer of 4 μm also served as a recess for enzyme membrane integration on the electrodes.

### Ceramic-based platforms

Ceramic-based in vivo sensors for the brain have been extensively developed and applied in the work by the group of Gerhardt. They were based on 0.127-mm-thick ceramic substrates with sputtered and lift-off patterned platinum metallization [28] (Fig. 2b). Thinner substrates (i.e. 25–50 μm) were too difficult to use. A 10-μm-thick polyimide layer served as the insulation. Needles were first shaped by rectangular dicing of the ceramic substrate, and triangular shapes were produced or tapering was done by laser cutting. A total length of up to 1 cm was reported, with the thickness increasing stepwise from 125 μm [28] or continuously for the triangular shape [25], with a sharp tip of 2–5 μm. Up to four working electrodes of various geometries have been realized (e.g. square 50 μm × 50 μm [28] or stretched to 15 μm × 330 μm [88]). On these microneedles, different amperometric biosensors for glutamate [28, 45], lactate [25], choline [30] and acetylcholine [38] have been realized. These sensors have been used in a large number of neuroscience studies on both anaesthetized [46, 89] and freely moving [47, 90] rodents.

By application of microfabrication technologies, needle-type in vivo sensors were successfully realized on ceramic substrates. As for silicon, brittleness limits the overall length and minimum thickness. The number of available processes, especially for etching into the depth of the material, is much larger for silicon though. Most likely, the thin ceramic substrate limits the reported size of the wafer/substrate (2.5 cm × 2.5 cm [88]), whereas in silicon, individual chips can be thinned out and standard wafer sizes (e.g. 100 mm diameter at 300 μm thickness) can be used. Dicing by blade of the ceramic substrate allows only limited geometric shapes, and additional, chip-level modifications such as laser cutting are needed for final shaping.

### Polymer-based platforms

The increase of biocompatibility and the ease of use of flexible, polymer-based platforms has is desirable for both in vivo sensors and neurotechnological devices. One of the primary limitations though is that unguided penetration into tissue (e.g. into the brain in a stereotaxic setup) is not possible for very thin probes. Most developments in this field have been for neural implants rather than biosensors. Still, an overview of the technologies applied may be helpful for biosensor development.

Polyimide resin can be spin-coated on silicon wafers and sputtered with platinum as electrode material, which is then insulated by another spin-coated polyimide layer [91]. Electrode opening and shaping of devices can be achieved by reactive ion etching. Electrodes as small as 10 μm and with a total thickness of less than 20 μm can be achieved. Polyimide in a precursor state can also be laminated on such structures to achieve additional layers [92]. As a shaft for an optical waveguide, including a microchannel for drug delivery into brain tissue of mice, 10 μm spin-coated polyimide as the substrate was combined with 160-μm-thick SU-8 permanent epoxy resist as the waveguide [93]. The SU-8 fluidic channel was glued to the back side. The concept of rigid penetrators, polyimide backed by silicon or metal, attached to integrated, flexible polyimide cables was demonstrated for neural implants [94, 95]. This principle is also potentially applicable to microbiosensors. An all-polyimide probe for impedance/resistivity measurement in the brain was developed [96]. On top of the spin-coated 20 μm polyimide substrate, platinum was sputtered and insulated by another 4-μm polyimide layer. Electrode opening and shaping of the shaft was achieved by dry etching. At a total length of around 30 mm and a width of 500 μm, the device had to be supported by a glass capillary for insertion. By the spin-coating of SU-8 onto a glass or silicon substrate and subsequent release, shafts for neural recording made entirely from SU-8 can be realized [97, 98]. Metal structuring on the SU-8 was done by platinum sputtering and lift-off or direct etching of gold and chromium. Up to five layers of 20-μm-thick SU-8 were stacked, carrying up to four 20 μm electrodes. The integration of a microfluidic channel for drug delivery was also demonstrated with this technology [99]. Recording of DC signals was combined with amperometric glucose and oxygen sensing [100]. The substrate was a spin-coated polyimide of 12 μm thickness, with platinum working and iridium oxide reference electrodes, which was spirally rolled to form a 0.7 mm diameter tube-like structure.

For the fabrication of glucose and lactate biosensors in pioneering work, we used a 100 μm polyimide film, vapour-deposited platinum metallization, and insulation by another 2 μm polyimide layer [101]. Two working electrodes and a Ag/AgCl reference electrode were included. Enzyme immobilization was by entrapment in poly(hydroxyethyl methacrylate)-based (PHEMA) hydrogels. Lactate sensors were later added on a modified device [21]. For glutamate, glucose, lactate and oxygen in vivo sensors for both the brain and subcutaneous applications, we used a polyimide film of thickness between 50 and 100 μm and insulated the platinum electrodes with 5 μm SU-8 epoxy resist on both sides [26, 44] (Fig. 2c). Additionally, we introduced a laminated, 38-μm-thick negative dry film resist as another layer. The main feature of this relatively thick material is that it forms an even deeper well around the electrode in comparison with that obtained with other methods [23, 102], which allows volume control during sensor membrane deposition. By the stacking of membranes, the control of sensitivity and linear range is possible for different enzyme immobilization techniques. The electrode well also provides protection and limits crosstalk. This hybrid approach combining thin-film and laminate technology yields a flexible sensor strip which is still able to penetrate brain tissue precisely without guidance up to length of 10 mm with a stereotaxic setup. A total device length of up to 50 mm is possible for specific surgical applications. The advantage here is that handling of the sensor is convenient because the laminate is durable and there is no risk of breaking. Also, the sensor can flex along tissue or auxiliary structures during implantation. Both the length and the flexibility/durability widen the field of application considerably, especially beyond use in the brain. Cutting of the sensors from the polymer film wafer by a drag knife allows efficient shaping of different geometries even after sensor integration and without expensive dry etching or exposure to harmful chemicals and heat. All together, this concept allows efficient mass fabrication of up to 76 sensors per wafer with relatively low cost compared with other microfabrication technologies. Of course, many of these advantages apply to flexible devices in general.

## Biosensor integration

### Enzyme immobilization and sensor performance

The immobilization of the enzyme and modification of the electrode are undoubtedly the most important steps in the construction of electrochemical biosensors. The enzyme needs to be permanently fixed to the electrode, without a major loss in activity, thus allowing a continuous and stable sensor performance. This can be achieved by different techniques, where the major concepts used for in vivo platforms are chemical cross-linking by an active cross-linker, often combined with a neutral matrix, entrapment in a hydrogel matrix, and integration into an electrodeposited polymer layer. Certainly, an abundance of other concepts and crossovers thereof exist, which cannot all be covered within this article.

The layer in which the enzyme is immobilized affects both sensor performance and biocompatibility and can thus be used to modify both. For very sensitive sensors (e.g. for the neurotransmitter glutamate), the goal is to immobilize a high density of active enzyme into a highly permeable membrane to achieve the maximum signal. A simple monolayer of enzymes is not enough as it is kinetically limited because of full catalytic occupation of the enzyme molecules. So, a thin, yet three-dimensional, matrix with high loading has to be used to generate a higher signal. The signal becomes highest at the transition between diffusion limitation and kinetic limitation. Therefore the more this matrix limits analyte diffusion, the more the signal will decrease again.

For a sensor with a high linear range, the aim is to achieve high oxygen diffusion compared with analyte diffusion, resulting in a fully diffusion-limited operation mode. In the enzymatic layer, the enzyme can be overloaded, which means its concentration is higher than needed, which makes the sensor less dependent on loss or degradation of parts of the enzyme. Together with an immobilization which traps and fixes the enzyme stably in the membrane, long-term stable sensors can be realized. Generally speaking, these implications mean that there is very often a trade-off between high signals and/or a fast response and a linear range and/or long-term stability. The parameters which can be realized by amperometric biosensors are limited to the available oxidase enzymes, at least for first-generation biosensors.

A comparison of in vitro long-term stability is difficult because either data are not presented in the studies or methods are not comparable. We believe long-term stability should be evaluated only under continuous measurement with the analyte present. Calibrations and storage in buffer in between measurements will most likely not yield the same results as continuous measurements. In sensors where hydrogen peroxide is produced, the exposure of the enzyme to this hydrogen peroxide contributes to the degradation of the enzyme and thus can lead to a decrease in sensitivity. Also, the local pH change due to the hydrogen peroxide oxidation at the electrode can play a role. Both depend on the local concentration of hydrogen peroxide, which of course depends on the analyte concentration, but varying proportions make comparisons difficult. The best indicator might be the absolute current density obtained.

For the in vivo situation, it is even more challenging to compare performance. In short-term experiments, calibration is mostly done before the experiment, and a certain loss in sensitivity is then accepted but not measured. To track the change of sensitivity in vivo, an in situ calibration scheme would be necessary. To make comparisons, it would also have to be standardized. However, in situ calibrations are not even possible for all parameters. For example, in the brain, the neurotransmitter glutamate is taken up rapidly from the extracellular space. Therefore injected reference concentrations clearly result in a much lower concentration being measured by the sensor [26, 28, 29]. Even for the different sensing methods, most importantly microsensors versus microdialysis, different measured absolute concentrations have been reported [103, 104]. More complex calibration and referencing schemes, such as comparison with blood values measured by clinical analysers, are also not possible for substances whose concentrations fluctuate quickly and locally, and are thus limited to mainly glucose and lactate. Comparisons with in vitro calibrations done after the in vivo measurements may suffer from effects of the explantation procedure and are even sometimes discouraged as misleading [25]. Maintaining the same interface as in vivo is also challenging because substances desorb from or leech out of the sensor membrane after explantation [105].

#### Cross-linking

The most straightforward method to immobilize enzymes is to chemically cross-link them to a carrier matrix. The most commonly used homobifunctional cross-linker is glutaraldehyde and the most commonly used matrix is bovine serum albumin. The two reactive groups of glutaraldehyde connect to both the enzyme and the matrix proteins. Usually, the enzyme is dissolved together with bovine serum albumin, and as the final step before deposition, glutaraldehyde is added. Deposition is often done by dip-coating, drop-coating or dispensing of microdroplets. Cross-linking occurs as the droplet dries, and additional membrane layers can be added later to increase the total amount of immobilized enzyme. A diffusion-limiting and/or selective membrane, such as Nafion or polyurethane, can be added on top later to increase the linear range or selectivity. This method allows a high enzyme concentration because of the low viscosity of the precursor solution. It has been applied for all types of microsensor platforms, including wires, fibres, and discs, and ceramic, silicon and polymeric substrates (Table 3). Frequently, it has been used for the fabrication of glutamate sensors, where usually the aim is sensitivity as high as possible because of the expected low in vivo concentration. Besides glucose and lactate, sensors for choline produced by immobilization of choline oxidase [29, 30] can also be realized. One can measure acetylcholine by additionally immobilizing acetylcholinesterase, which converts acetylcholine to choline [38]. The choline background must be subtracted with use of a dual electrode. Recently, an adenosine sensor was demonstrated that uses the dual electrode principle with three different enzymes, where xanthine oxidase produces the hydrogen peroxide [39].

**Table 3:** Comparison of enzyme-based amperometric in vivo microsensor performance (in vitro) across different platforms. Highly sensitive glutamate/choline sensors, lactate sensors with a wide linear range, and performance data for the intermediary product hydrogen peroxide are included. Where no specific values are stated, they were calculated from the data shown and may therefore be approximations. The achieved sensitivity, limit of detection (*LOD*) and linear range are stated for a given platform, the enzyme immobilization method used, the electrode material and the interference rejection method

Parameter	Electrode	Substrate	Enzyme membrane	Sensitivity (nA mm^−2^ μM^−1^)	LOD (μM)	Linear range (mM)	Interference rejection	References
Glutamate	Pt/Ir	Wire	GA + BSA	0.26	-	-	Nafion	[27]
	Pt	Wire/disc	BSA + PPD	0.32		0.1	PPD	[83]
	Pt	Silicon	GA + BSA	0.5	0.79	0.08	PPy + Nafion	[106]
	Pt/Ir	Wire	GA + BSA	0.88	-	-	x + AAOx	[36]
	Pt	Silicon	GA + BSA	0.95	0.42	0.1	PPD	[29]
	Pt	Wire	GA + BSA	1.03	-	-	PPD + Nafion	[107]
	Pt	Silicon	GA + BSA	1.52	0.32	0.3	PPy + Nafion	[42]
	Pt	Polymer	GA + BSA	2.16	0.22	0.15	PPD	[26]
	Pt	Ceramic	GA + BSA	2.23	0.52	0.8	PPD	[45]
	C	Fibre	PEGDE + RH	0.32	-	0.1	AAOx	[33]
	C	Fibre	PEGDE + RH	0.82	0.09	0.1	AAOx	[40]
	C/Ru	Fibre	GA + BSA	2.39	-	-	Nafion	[22]
H_2_O_2_	Pt	Ceramic	None	1.76	0.27	-	Nafion	[28]
	Pt	Polymer	None	3.8	<0.1	1	PPD	[26]
	Pt	Silicon	None	3.85	-	0.06	PPD + Nafion	[107]
	Pt	Ceramic	None	4.88	0.13	-	None	[28]
	Pt	Polymer	None	8.92	<0.1	1	None	[26]
Lactate	Pt	Ceramic	GA + BSA + PU	0.008	78	20	PPD	[25]
	Pt/Ir	Wire	GA + BSA + x	0.01	-	5	x	[37]
	Pt	Polymer	PHEMA	0.011	-	20	PPD	[21]
	Pt	Polymer	PHEMA	0.021–0.256	2–15	1–10	PPD	[26, 44]
	C	Fibre	GA + BSA	0.009	-	-	CA	[41]
Choline	Pt	Silicon	GA + BSA	1.32	0.3	0.3	PPD	[29]
	Pt	Ceramic	GA + BSA	2.64	0.412	0.2	Nafion	[30]

*AAOx* ascorbate oxidase, *BSA* bovine serum albumin, *CA* cellulose acetate, *GA* glutaraldehyde, *PEGDE* poly(ethylene glycol) diglycidyl ether, *PHEMA* poly(hydroxyethyl methacrylate), *PPD* polyphenylenediamine, *PPy* polypyrrole, *PU* polyurethane, *RH* redox hydrogel, *x* not specified

If we compare glutamate sensor performance data (Table 3), the best example for high-sensitivity biosensors, the maximum achievable sensitivity across different platform seems to be somewhere around 2 nA mm^−2^ μM^−1^. This corresponds to roughly the same enzyme loading into the precursor solution at around 100–200 U ml^−1^. A higher concentration leads to issues with solubility and too high viscosity. A thicker membrane and thus higher absolute amount of enzyme means diffusion limitation of the glutamate into the membrane because of the increasing thickness. A future substantial increase in sensitivity is unlikely without a radical change in the approach. Addition of an electropolymerized layer on top, either for interference rejection or for fixing of the enzyme, leads to a decrease in sensitivity. The limit of detection for this principle was repeatedly found to be several hundred nanomoles per litre (Table 3). Noise in and limited resolution of the signal due to the low currents, as well as drift, play a role here. The choline sensors, for which the immobilization is similar, show performance data comparable to those for the glutamate sensors (Table 3). The response time for this method is short and typically in the range of 1–5 s.

From a comparison of the linear range, it is obvious that highly sensitive sensors have only a limited range of a few hundred micromoles per litre, the same concentration range as that of the available oxygen. It is also apparent that for sensors where the linear range needs to be extended (i.e. the lactate sensors in Table 3), sensitivity must be sacrificed. Also, the criteria for linearity must be taken into consideration when one is comparing sensor performance. We observed that by choosing a different *R*^2^ (e.g. 0.997 instead of 0.999), we could easily change the declared linear range by a factor of 3. In practice, the dip-coating or drop-coating of thin polyurethane layers on top of the enzyme membrane is a common method to increase the linear range [23–25]. It is also possible to electrodeposit the enzyme from a solution onto the electrode by application of a positive potential, which attracts the negatively charged protein [29, 108]. The enzyme needs to be fixed by cross-linking or entrapment under another membrane afterwards.

Where data are available, they show that the oxidation of hydrogen peroxide at the platinum electrode is not or should not be the limiting factor for sensitivity, because higher current densities for hydrogen peroxide can be achieved with a good electrode quality (Table 3). Performance across the systems is in the same window, and addition of a selective membrane decreases the sensitivity. Still, the sensitivity for hydrogen peroxide is not magnitudes higher, so it must be considered in the design and performance evaluation of such sensors, especially in combination with deposited membranes.

A drawback of the cross-linking method is the limited sensor lifetime. A decrease in sensitivity under continuous operation of several percent per day is not uncommon [26]. Continuous long-term data are rarely shown though. Sensors are operated near the kinetic limitation of the enzymatic reaction; therefore a loss in enzyme activity immediately leads to a loss in signal. The immobilization has been reported to be stable though, in the sense that sensors can be stored in buffer without loss in sensitivity for times on the order of 1 month. For longer storage times, reduction in sensitivity was observed, underlining the limitations of this immobilization method [26, 29]. Reduction of the specificity of the enzyme by the cross-linking has also been reported, an issue that is rarely ever discussed [109]. For glutaraldehyde, a cross-sensitivity to other amino acids was found, and poly(ethylene glycol) diglycidyl ether was recommended as a milder linker, leading to better specificity. Other work found the glutaraldehyde method to be sufficiently specific [104].

#### Hydrogels

For the entrapment into hydrogels, the enzyme can be dissolved in an aqueous solution, alongside the hydrogel monomers, cross-linkers, plasticizers and the photoinitiator. This liquid precursor solution can be dispensed onto the electrode or the electrode can be coated with it. On UV exposure, the photoinitiator starts polymerization connecting the monomers and the cross-linker. Spatial photopatterning of the hydrogel is also possible [21]. With PHEMA, glucose, lactate and glutamate and glutamine sensors were realized [21, 26, 44, 110, 111]. By the stacking of different layers upon each other, diffusion-limiting membranes without an enzyme can be introduced, which are used to expand the linear range of the sensor (Table 3). By variation of the thickness of the enzyme membrane and/or the diffusion-limiting membranes, the sensitivity and linear range can be adjusted over a very wide range without compromising sensor performance parameters other than the response time. Even though glutamate oxidase can be immobilized this way, the concentration of the enzyme cannot be high enough to achieve highly sensitive signals comparable with those obtained with the cross-linking method.

The PHEMA hydrogel stabilizes the enzymes very well, regarding both the natural conformation and thus activity and the confinement/fixation of the enzyme inside the membrane, and therefore leads to long-term stable sensors, particularly for otherwise difficult to immobilize lactate oxidase [26, 110]. Besides, the PHEMA membrane is mechanically durable and stable, and maintains unaltered integrity throughout storage in both buffer solutions and a dry environment for years, and enzyme activity remains. It also has high hydrophilicity and shows little protein adhesion, which results in high biocompatibility in complex environments, both in vitro and in vivo. Limiting factors are the limited solubility of the enzyme in the precursor solution and its high viscosity, which limits the enzyme concentration and makes nanolitre-scale dispensing or drop-coating the only feasible way to bring the membrane onto the electrodes.

The bienzyme, second-generation method using a redox hydrogel [112, 113] was also realized for glutamate [33, 40, 85]. Glutamate oxidase was wired to the redox hydrogel containing an osmium complex by the linker poly(ethylene glycol) diglycidyl ether. The second enzyme was horseradish peroxidase. Ascorbate oxidase was also co-immobilized to improve selectivity [114]. The performance was comparable to that of the first-generation biosensors (Table 3), although response time at more than 20 s was slower [40], most likely due to diffusion limitation by the dense gel. The hydrogel was applied to carbon fibres by dip-coating. Not dependent on the hydrogen peroxide oxidation at the electrode, the applied potential can be lowered to −150 mV (vs Ag/AgCl) [40]. The method was also applied for choline and acetylcholine sensors [115].

#### Electrodeposited polymers

Besides their use as permselective layers to reject interferents, electrodeposited polymers can also be used to immobilize enzymes. These thin polymer/composite membranes in the range of 10 nm can entrap the active enzyme on the electrode and block interfering substances at the same time [116]. In vivo sensors were primarily realized for glucose [117]. It is questionable whether these thin membranes can immobilize enough enzyme for high-sensitivity neurotransmitter sensors. Another option is to dip the electrode into a solution containing the enzyme and bovine serum albumin, and deposit the electropolymerized layer afterwards to fix the enzyme in place. This method was applied for glutamate on platinum wire and disc electrodes [82, 83, 118]. The sensitivity was lower than for the cross-linking method (Table 3).

### Interference rejection principles and selectivity

One of the key parameters of sensor performance is selectivity. Typically, selectivity means a high signal caused by the measured analyte, compared with a low signal caused by the unspecific background. Selectivity is particularly important for in vivo application. Analyte concentrations are often low or change rapidly over time. Also, a variety of other substances are usually present, often in an unknown concentration and composition. Electrochemical sensors mostly rely on the conversion of a substance at the electrode, where the current generated is proportional to the analyte concentration. Either the analyte itself is converted or an intermediate product is converted. In the case of enzyme-based biosensors, very often this intermediate substance is hydrogen peroxide. Thus to ensure selectivity, the reaction of interfering substances at the electrode must be prevented.

One method is the applied electrochemical protocol itself, which means the polarization of the electrode to an appropriate potential. Most electrochemical sensors are based on the oxidation of the analyte or the intermediary product. On noble metal electrodes, in a potential region where, for example, hydrogen peroxide is oxidized, a large number of biogenic substances are also oxidized, producing a comparable or at least non-negligible exchange current density. These are most prominently ascorbic acid, which is present in a relatively high concentration in the brain [119], uric acid or catecholamine neurotransmitters such as dopamine [7]. They can also be certain drugs (e.g. acetaminophen). In redox-hydrogel-based sensors, the applied potential can be kept lower (usually around −100 mV vs Ag/AgCl) than for hydrogen peroxide oxidation (+400 to +700 mV), which also reduces cross-sensitivity [33]. The major reducible species in the biological environment is oxygen, which is of course present in most tissues under physiological conditions. The overpotential for its reduction is, however, high enough on most common electrode materials that no reduction occurs in competition with a desired oxidation reaction [120, 121].

Another widespread method to increase selectivity is the use of a permselective membrane, an additional layer on the electrode, which prevents interfering substances from reaching the electrode but allows the transport of the analyte or intermediary product. The commonest methods here are thin polymer membranes, with their effect based on size exclusion or charge exclusion (e.g. electropolymerized aminobenzenes or drop-coated Nafion), where small, uncharged molecules such as hydrogen peroxide can pass through and large interferents are rejected because of their higher molecular weight or charge.

The most frequently used permselective membranes are electropolymerized polymers. The monomers are dissolved and then anodically deposited onto the electrodes, often by cyclic voltammetry. Electropolymerized poly(*o*-phenylenediamine) and poly(*m*-phenylenediamine) were introduced as antifouling layers based on size exclusion [32] and have been used since. They were used for the coating of platinum electrodes on polymeric substrates [21, 26, 44], ceramic-based sensor platforms [122] and wires [107] and carbon fibres [115], deposited either by cyclic voltammetry or by constant potential (Table 3, interference rejection). They can also be used to immobilize the enzyme in a selective polymer/enzyme composite layer. The deposition on top of the enzyme layer was shown on silicon-based devices [29]. Here, the efficiency was demonstrated for glutamate and choline sensors, but the question remains how the analyte efficiently reaches the enzyme if the blocking layer is on top of the enzyme membrane. Electrodeposited polypyrrole [43], polyphenol [123] and the lipid phosphatidylethanolamine, applied by dip-coating [82], can be used as other size-exclusion layers. Nafion can serve as a permselective membrane based on charge. It was dip-coated on wires [27], carbon fibres [33], ceramic substrates [28] and silicon [43], where it also acts as a diffusion-limiting membrane. Different permselective membranes for glutamate sensors based on platinum wire electrodes were reviewed by Wahono et al. [107], among them poly(*o*-phenylenediamine) and poly(*m*-phenylenediamine, polypyrrole, and polyaniline, also in combination with Nafion. The best performance for interference rejection was found for poly(*m*-phenylenediamine) deposited by cyclic voltammetry. We also found this material to be ideal. Not only does it reject large molecules very well, it also does not limit hydrogen peroxide diffusion too much. In our case, hydrogen peroxide transport was still half of that on an unmodified electrode [26]. The process itself allows homogeneous coating of a large number of electrodes in parallel, because of its self-limitation. It exhibits good stability, although continuous exposure to hydrogen peroxide will lead to degradation of the layer over days.

To determine the performance of permselective layers, selectivity is often measured by the ratio of the sensitivity for the analyte to the sensitivity for the interferent. However, if the electrode sensitivity for the interferent is inherently low, this ratio becomes high. Thus it can be helpful to determine the sensitivity for the interferent on the unmodified electrode first and then compare it with the sensitivity obtained with the permselective membrane to directly evaluate its effect. Also, it has to be considered that the interferent (e.g. ascorbic acid) may react with hydrogen peroxide when the analyte and interferent are measured together. Therefore we found it practical to measure the interferent alone first.

The specific nature of enzymes is obviously the reason why they are used as the sensor recognition element on the working electrode and they therefore inherently contribute to the selectivity. The immobilization of a second enzyme which breaks down typical interferents is another way to further increase selectivity. Ascorbic acid oxidase immobilized on top of the sensing enzyme membrane to break down ascorbic acid from the brain has been frequently used along with glutamate or glucose oxidase for this purpose [27, 33, 36, 85].

Finally, the blank electrode principle is another way to increase selectivity. A second electrode, ideally with the same membrane setup as the working electrode, only without the enzyme, is implemented. The current of the blank electrode is then subtracted from the signal of the working electrode [26, 28, 29, 110]. With this method, it is possible to reduce not only chemical interference but also long-term effects on background currents (e.g. due to electrochemical processes in the electrode material [124]), electrical noise coupling into both electrodes or general effects such as offsets due to changes in temperature or humidity. Particularly in the in vivo application, with a number of often unknown and fluctuating background signals present, the implementation of a blank electrode can be very useful.

## Biocompatibility

Without doubt, biocompatibility is and remains one of the primary aspects and grand challenges for in vivo sensing. Despite its importance, within this scope, we can give only a short overview of the topic and general perspectives for microsensors. The term “biocompatibility” itself has been defined and refined to a great extent [125–127], so we will simply regard it as the interaction between the organism and the sensor device which affects the functionality of the sensor. One of the main reasons why biocompatibility is so important for chemical and biosensors is that there is quantitative exchange of molecules, not only charge, across the interface between the sensor or sensing element and the organism. This transfer and the resulting sensor signal are in most cases highly dependent on diffusion. Therefore changes at the interface—these can encompass changes in the surrounding tissue, adsorption or deposition of molecules at the interface or even permeation into the membrane—will affect sensor performance. The first goal must therefore be to achieve as little change at the interface as possible.

Commonly, the reactions between the sensor and the host organism are divided into different phases. First, within a few seconds to hours, molecules, proteins or fragments thereof adsorb to the sensor surface [105, 128]. A substantial decrease in sensitivity during this phase has been reported, especially for glucose. The exact reason for this decrease is still under debate. The effect might not be as severe in a less demanding environment (e.g. the brain) or for smaller analytes, for which the diffusion is not limited as much. Experiments in the CNS are often short term, so long-term stability has played a minor role here to date. Molecules or fragments may migrate into the sensor membrane, further reducing its permeability. Some of the adsorption effects or biofouling is also reversible. When one is comparing in vitro calibrations done before and after the in vivo measurements, this subject must be considered. Over the following days, a complex cascade of foreign body reactions will be triggered, which includes the accumulation of certain types of molecules and cells near the implant as part of the inflammation reaction. This can lead to further impairment of sensor function. Finally, an encapsulation of the implant will occur, a phase where sensor performance is severely affected but which in many cases is beyond the sensor lifetime.

To counter the above-mentioned issues, two main concepts for sensor interfaces are pursued. These are the passive coating of the sensor interface with a multitude of different materials, or the active release of substances from the sensor membrane material to mitigate the foreign body reaction. All sensors shown in Table 3 feature the passive method. The polymer membranes immobilizing the enzymes or ensuring selectivity act to increase biocompatibility, mostly from the sensor perspective in terms of maintaining sensitivity and prolonging operation. So far, little effort has been put into trying to coat the entire device with a more biocompatible layer to increase overall biocompatibility. Magnetron-enhanced plasma-polymerized nanofilms are prospective candidates, as they allow batch processing at room temperature after sensor integration [129]. Among the active methods, the release of nitric oxide from the sensor membrane is a promising concept to increase the long-term stability of glucose sensors [123, 130–132]. The location of placement for all in vivo sensors has almost exclusively been the interstitial fluid, not the bloodstream. Because of its complexity, blood provides a harsher and more difficult environment for the sensor. A sensor in the bloodstream also poses a risk for the host organism because of its thrombogenicity. If sensors in the bloodstream are desired, future work must solve both challenges.

Furthermore, it has become more and more evident that true long-term biocompatibility means sensor flexibility and/or elasticity must be matched with that of the surrounding tissue [133]. Obviously, for metal needles or probes made of materials such as silicon or a ceramic, but also for most polymers, such as polyimide, the elastic modulus is still several magnitudes higher than that of tissue, especially that of the CNS [134]. Of course, the main reason why microsensors are still more or less rigid and much stiffer than tissue is that they need to be inserted into the said tissue, preferably without any guiding structures and with high precision. Yet, more effort can certainly be directed towards the integration of biosensors on highly flexible/elastic materials and the design and fabrication of such in vivo platforms, as well as towards the development of new sensor device placement or insertion concepts (e.g. dissolving inserter vehicles come to mind). With increasing long-term stability of biosensors, this topic of structural biocompatibility might become even more important.

## Conclusions

Having discussed the different aspects of application, microfabrication, sensor integration and performance of miniaturized amperometric in vivo biosensors, we wish to express our concluding thoughts and outline future tasks for the various topics. In doing so, we wish to address the engineering perspective as well as the application perspective.

Microtechnology offers a multitude of possibilities to fabricate and integrate microsensor platforms for in vivo application. Particularly in silicon technology, new platforms have emerged, based on recently optimized fabrication pathways. Wafer-scale processing, including reference electrode integration, and even membrane/sensor integration can mean an efficient mass fabrication of ready-to-use devices. The development of new sensing principles, however, is often done on a macroscopic scale. There must be a translation of these principles to usable platforms so that sensors do not share the common fate of remaining “laboratory curiosities” [135]. The amperometric in vivo biosensors discussed here all employ fairly traditional sensing principles. Thousands of new enzyme immobilization concepts and electrode materials are developed that have surprisingly little impact on sensor performance. Many basic sensors are developed without even an attempt being made to solve a performance problem or without the opportunity being offered to apply them on micro platforms. The relatively conservative in vivo sensor principles also show that the performance needs to be proven and characterized well before in vivo use, and the platform needs to be very reliable. The challenge clearly lies in the complete package from platform design and sensor integration to a feasible applicability.

With the available technology, platform sizes are still considerably larger than the cellular dimension. Accessing synapses or even the intracellular space lies way in the future. Future trends may go in this direction. Whether this development includes enzyme-based electrochemical biosensors is questionable. They are powerful tools, yet there are number of inherent disadvantages. An appropriate enzyme (or enzyme system) must exist, and it must be immobilized in a stable way. There is often a trade-off between sensitivity and quick response, and long-term stability. If the enzymatic reaction requires oxygen, a dependency is to be expected if oxygen supply is restricted. Diffusion occurs to and through the sensor membrane, so all signals are diffusion limited, with diffusion coefficients constant by nature.

We did a comprehensive performance comparison for enzyme-based amperometric in vivo microsensors (Table 3). The key performance data, such as sensitivity, linear range and detection limit, fall in a relatively small range, even across platforms, electrode materials and immobilization methods. Sensitivity of around 2 nA mm^−2^ μM^−1^ and a detection limit below 1 μM are feasible. The “selective membrane/platinum/hydrogen peroxide/oxidase enzyme” system seems to be fairly optimized, with no obvious room for improvement. Increasing the linear range means limiting diffusion and thus sensitivity. Aside from the available and well-used oxidase enzymes, future work may focus on new combinations of enzymes and multielectrode approaches to realize new parameters. Engineering of the membrane deposition process itself offers a multitude of options and reproducibility and exact tailoring of the sensitivity/linear range can be challenging. More long-term data from continuous measurements are desirable especially for highly sensitive sensors outside the glucose world, both in vitro and in vivo. This can include comparisons of in vitro calibrations done before and after the in vivo measurements or in situ calibration as well as the identification of adsorbing substances. The determination of sensor parameters such as sensitivity, linearity, selectivity, and long-term stability as well as the exact methods applied to determine these parameters should be communicated clearly and paid attention to. More harmonization in this regard would be useful. The same is true for in vivo data.

Biocompatibility remains a crucial topic. In vivo amperometric biosensor use is still limited to a few days. Passive tailoring of the interface and the active release from the sensor both leave immense room for future developments. Still, there seems to be no single optimal strategy. Also here, more work outside the field of glucose sensing would be useful. Further, the controlled interaction of the sensor with the host organism should be expanded from the mere interface to the entire device. More detailed investigations of the actual compounds which are responsible for sensor degradation are desirable. For quantitative long-term measurements, in vivo calibration schemes are inevitable. Flexibility of the devices is an underrepresented aspect regarding structural biocompatibility. The process of insertion is a challenge for flexible devices.

Potentiostats and instrumentation are essential for amperometric sensor operation. Advanced electrochemical protocols require more sophisticated instruments, which cannot be self-made easily and which are different from those in neural recording. Commercially available instruments are often large and expensive. Measurements on freely moving animals require headstages, which are often available only for proprietary sensors. The combination of disposable microsensors and miniaturized electronics leaves room for improvement. With regard to portable or wireless sensors as well as multichannel sensors, technological developments in this direction are necessary.
